# An interview with Kazuo Tanne

**DOI:** 10.1590/2176-9451.19.1.010-018.int

**Published:** 2014

## Abstract

Professor Kazuo Tanne has a degree in Dentistry by the University of Osaka where he also received the title of PhD in Orthodontics. From the 1^st^ of July, 1993 to the 31^st^ of March, 2013 he was the head of the Department of Orthodontics and Craniofacial Development Biology at the University of Hiroshima, in Japan. Professor Tanne has been the head of the Japanese Association of Cleft Lip/Palate, and nowadays is the head of the Asian Orthodontic Society which comprises 18 orthodontic societies in the Asia/Pacific area. He has published more than 700 articles and more than 60 books and/or chapters about many different topics. Professor Tanne has conducted interesting researches that focus on temporomandibular disorders, tooth cryopreservation, cleft lip/palate, molecular/cell biology and genetic engineering for bone repair. I am honored to say that I was advised by Professor Tanne during my Doctorate in Orthodontics between 2006 and 2010. During that period, I had the opportunity to fulfill my expectations towards the excellence of education provided by the University of Hiroshima as well as by Professor Tanne who also proved to be a great human being with a noble heart. We have become great friends and fortunately I had the chance to learn much more than Orthodontics. Tanne Sensei, as he is known in Japan, has a deep admiration for Brazil and the Brazilian culture. He has been in Brazil in three occasions, when he made friends in many different locations.

Emanuel Braga Rêgo

**Figure:**
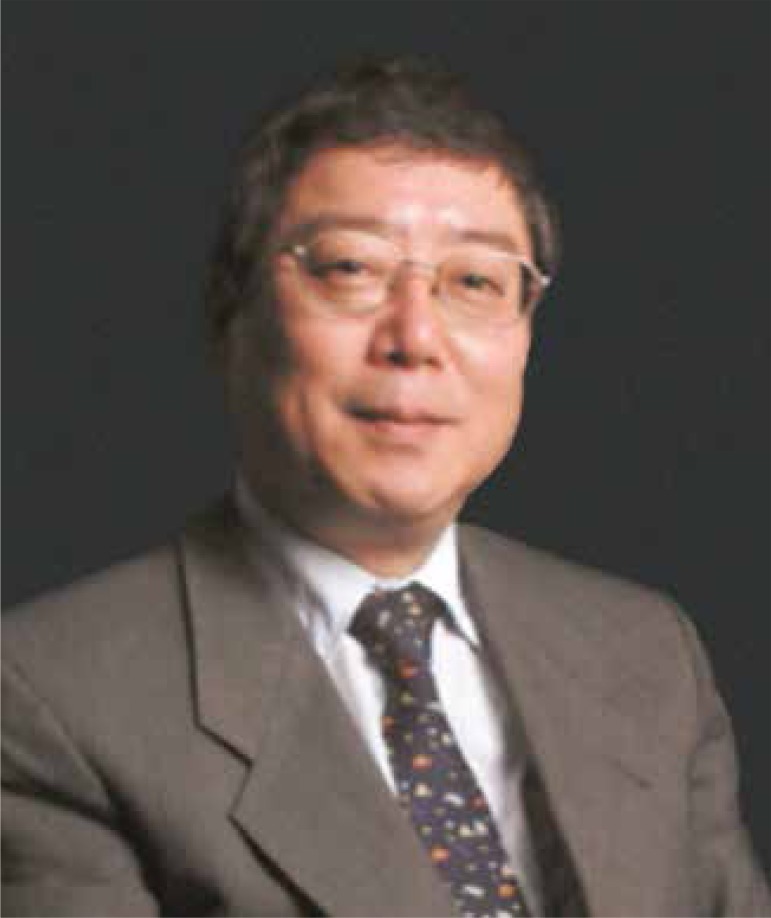


## Professor Tanne, you have been in Brazil on three different occasions during when you had the chance to attend conferences and exchange experiences with renowned Brazilian universities such as the Federal University of Rio de Janeiro (UFRJ), State University of São Paulo (UNESP) in Araraquara, State University of Campinas (UNICAMP) in Piracicaba and the University of Brasília (UnB). Additionally, you have advised six Brazilian orthodontists in the postgraduate program of the University of Hiroshima. In this context, what are your impressions of Brazilian Orthodontics? (Emanuel Braga Rêgo)

I have been to Brazil three times to attend scientific events. Among these events, the 49^th ^International Dental Meeting, in Araraquara, was the most impressive, given that it was my first time in Brazil. Moreover, I had the opportunity to teach a one-week course for some dental students at UNESP, which was highly advantageous to them. In this opportunity, I have to admit I found the knowledge of Brazilian students to be a little inferior in comparison to the Japanese, especially in regard to scientific research. After that, I attended to other events: One was held in Campinas, in April, 2006, during which I had the opportunity to teach a course to the students of Orthodontics at UNICAMP, whereas the other one was held in Fortaleza, in September, 2009 (CLEFT). After these two experiences, I noticed that students' level of knowledge had increased substantially in clinical and scientific terms. Nevertheless, I would like to suggest that all orthodontists, especially those involved with education, promote basic researches that can turn into useful evidence for the development of new therapeutic systems and techniques available for day-to-day Orthodontics. I also would like to highlight the importance of scientific exchange with other countries as a practical and quick way to produce the best performance in research. Incorporating young orthodontists/scientists who have received a doctorate title or have attended training programs abroad into Brazilian universities is undoubtedly a very positive measure.

## Professor Tanne, could you briefly explain the educational process students go through in Japan in order to become an orthodontist? (Emanuel Braga Rêgo)

Before explaining the educational process, I would like to present the current status of Orthodontics in Japan. The Japanese Orthodontic Society (JOS) is the official association in Japan. Up to February, 2012, it had 6,375 members. We can make an estimate based on the general population in Japan, which is of approximately 120 million people. The number of colleges authorized to offer postgraduate courses in Orthodontics is 29. The number of orthodontists certified by the JOS is 2,739 or 43% of all members (JOS members do not necessarily hold a certificate of Orthodontics). The number of orthodontists who hold a specialist certificate (a title that is similar to the Board certification) is 298, which corresponds to 5% of all members or 11% of all certified members. In other words: In Japan, the orthodontic certificate issued by JOS requires a 5-year postgraduate course and finishing of 30 cases, out of which two are selected for an oral test. Subsequently, there is a more specific certification that is called specialization: The applicant must present ten cases that have been treated with at least two years in retention and, then, undergo another evaluation. Only those who hold an orthodontic certificate issued by JOS are allowed to apply for the title of specialist.

## Professor, Hiroshima is well known in Brazil for the atomic attack it underwent during the Second World War. After this interview, at least for the orthodontic community, Hiroshima will also be known for its excellence in orthodontic education and scientific production. Could you highlight the most relevant topics that are being studied at the University of Hiroshima nowadays? (Emanuel Braga Rêgo)

Since I have been promoted to head of the Department of Orthodontics in Hiroshima, I have always tried to educate young students with clinical training and clinical thinking towards research. Hence, I have always required that we develop relevant scientific topics, even though they wished to act as clinicians, only. As a result, three students have become professors and head of their respective departments in other universities, 81 students have received a PhD title whereas 102 have received the orthodontic certificate. The topics we are currently studying are as follows: 1) biomechanical, biological and molecular mechanisms, as well as therapeutic principles, of temporomandibular joint osteoarthritis; 2) cryopreservation of cells and tissues used in the teeth bank as well as for tissue regeneration in the treatment of bone defects in cleft palate patients; 3) root resorption prevention and repair with therapeutic low intensity ultrasound; 4) association between malocclusion, breathing and sleep disorders, evidencing that orthodontic treatment can improve one's quality of life; 5) application of amelogenin, a protein of tooth enamel, to repair tooth-surface defects.

## Initially, I would like to compliment you for the excellent job you have done in education and for the important researches you have developed at the University of Hiroshima, which greatly contributes to the development of Orthodontics. I would also like to compliment the Dental Press Journal of Orthodontics for the excellent choice for this interview. In Brazil, we have reference centers for the treatment of cleft patients. These centers have a multidisciplinary approach, i.e., they work with dental, orthodontic, surgical and psychological treatments. Specifically with regard to orthodontic treatment of clef patients, I would like to ask: 1) Can these patients receive orthodontic treatment in private clinics, i.e., not in reference centers? 2) Briefly, what is the logistics for these patients in Japan? (Fábio Lourenço Romano)

In Japan, the treatment for cleft lip/palate patients is covered by health insurance; therefore, they only have to pay for 30% of the costs of surgery and orthodontic treatment. Additionally, only certified orthodontists are allowed to treat cleft lip/palate patients, whether in hospital or specialized private clinics. On JOS website, there is a list of certified orthodontists and patients have free access to this information. With regard to treatment, the general protocol requires that patients be pre-surgically treated with the Hotz appliance. Afterwards, cleft lip and palate cosmetic surgeries are carried out at the age of 3 or 4 months old as well as at 18 months old. Orthodontic treatment is performed immediately after treatment for maxillomandibular discrepancy and anterior/posterior crossbite repair. At around 9 years old, during initial orthodontic treatment usually performed before eruption of permanent canines, secondary bone graft is carried out to repair the bone defect in the cleft area. Finally, once permanent dentition is complete, patients undergo orthodontic treatment with fixed appliances in order to have their dental arches lined up, in addition to attaining the most satisfactory occlusion. In this phase, orthognathic surgery may be proved necessary for asymmetry and severe bone discrepancies repair. Dental agenesis is corrected by means of fixed prostheses or implants placed after body growth is complete.

## Rapid maxillary expansion associated with protraction of the maxilla is one of the treatment methods employed for anteroposterior maxillary deficiency in growing patients,especially in cleft patients. Based on your experience, do you consider this treatment protocol efficient and stable? (Fábio Lourenço Romano)

Rapid maxillary expansion is very useful for posterior crossbite correction. However, before expansion, it is necessary to diagnose the anteroposterior maxillomandibular relationship. Should significant discrepancy be found between the bone bases, crossbite will necessarily be present, even if the transverse dimensions of the maxillary dentition are normal and the apical base is within normal standards and in harmony with the mandible. In this context, overexpansion of the maxilla is unstable or inconvenient for future treatment with fixed appliances. Based on the aforementioned considerations, we initially treat the anteroposterior relationship of children with orthopedic appliances, whereas adult patients undergo distraction osteogenesis surgery (assessed from 12 years old on).

## Do you believe that autograft of mesenchymal stem cells associated with hydroxyapatite carbonate will be successfully used for bone regeneration of cleft palate in the future? (Fábio Lourenço Romano)

I appreciate your interest in bone regeneration with mesenchymal stem cells, since it is a relevant topic about which we have been studying in our department at the University of Hiroshima. Firstly, we conducted an *in vitro *study to understand the proliferation and differentiation of these cells and also to assess whether these abilities can be controlled by addition of some chemical mediators such as cytokines and growth factors. Afterwards, we conducted *in vivo* studies. Artificial defects were made to the parietal bone of growing rats and mesenchymal stem cell cultures were transplanted to these defects. After 8 and 16 weeks, bone regeneration and sutural structures were noticed. Additionally, we conducted a similar experiment with Beagle dogs in which bone defects were made to the maxilla so as to simulate cleft palate. The latter was posteriorly filled with mesenchymal stem cells and hydroxyapatite carbonate. The results demonstrated prominent bone regeneration in the bone defects. Finally, we were able to prove that mesenchymal stem cells cryopreserved with the new freezing system (freezer with magnetic field) can also be successfully used for bone regeneration.

Based on these studies, we are confident that mesenchymal stem cells transplantation will become a great tool for the treatment of bone defects in cleft palate patients. Moreover, it is evident that the psychological stress to which patients are subjected due to the several surgeries they have to go through will certainly be minimized with this bone regeneration therapy. In our university, clinical trials have been made with regard to the use of this therapeutic method in cleft palate patients.

## It is a great pleasure and an important opportunity for me to ask a few questions to you, Professor Tanne, a renowned scientist who studies the pathophysiology of the temporomandibular joint. The identification of clinical signs is very important to diagnose temporomandibular disorders (TMD), however, such information is not always well understood by the clinician. In addition to TMD clinical signs, which methods have proved to be the most efficient in the diagnosis, treatment and follow-up of orthodontic patients with temporomandibular disorders at the University of Hiroshima? (Rogério Lacerda dos Santos)

TMDs are known as multifactorial diseases, therefore, a differential diagnosis with appropriate methods is required. Furthermore, we have to pay special attention to the fact that orthodontic patients have a considerably high prevalence of TMD, and do not usually identify the problem before orthodontic treatment onset. A differential diagnosis of TMD is generally done by means of clinical, functional and imagiological examinations. Additionally, modern biochemical tests are available, especially for patients with suspected temporomandibular joint osteoarthritis.

As for the diagnosis of TMDs, the clinical examination firstly aims at tracking the following four main symptoms: joint pain, noise, muscular sensibility and difficulty in mouth opening. With regard to noise, crackling must be differentiated from clicking for an appropriate diagnosis. A click generally suggests anterior disc displacement with reduction. Crackling, on the other hand, suggests a pathologic condition that is more severe than clicking, and it is associated with anterior disc displacement without reduction. Difficulty in mouth opening is established when the degree of opening is less than 35 mm for adolescents and less than 40 mm for adult patients.

Should any sign or symptom be detected, the patient is subjected to the functional examination. For this examination, occlusal patterns such as occlusal force, tooth contact area, muscle activity and fatigue as well as condylar movement, are analyzed. Among these patterns, the analysis of condylar movement is the most valuable for TMD diagnosis in terms of intra-articular pathology identified by the pattern and course of the condylar movement. At our clinic, the axiograph and a 6-degree jaw tracking device are used to analyze condylar movement. The use of these devices allows us to identify four different movements. If the condylar course is smooth and the linear length is greater than 10 mm, it will be considered normal. The 8-shaped pattern represents the crossing between the courses of opening and closing, which reflect the beginning of clicking noises between the disc and the condyle. Therefore, 8-shaped pattern sagittal condylar movement suggests anterior disc displacement which is divided into early, intermediate and late, according to the crossing point of the course that is affected by the degree of disc displacement. Late disc displacement is considered more difficult to treat due to being more severe. The other types of disc displacement are more restrict or mixed, and are generally related to severe pathologies and internal joint disorder.

Imagiological exams such as tomographies and magnetic resonance are recommended for patients with suspected joint internal disorder. Tomography imaging is useful to measure not only the joint spaces, but also the condylar position in the glenoid fossa. Additionally, it is used to detect condylar bone disarrangements. As for magnetic resonance, it is able to assess the level of displacement and the arrangement of the disc. At our clinic, the criterion proposed by Wilkes has been used for a differential diagnosis of internal disorders. The status of intra-articular pathology is classified into six stages. Stage zero: normal; stages I and II: mild disc displacement with reduction. It is important to highlight that no other significant mechanical symptoms can be recognized, except for the reciprocal click which is observed in stage I of the pathological status. From stage III to V, both disc displacement without reduction and condylar deformity can be observed.

It has been proved that the majority of TMDs can be diagnosed by means of several exams; therefore, I recommend that all aforementioned steps be taken in order to achieve a differential diagnosis and, thus, prevent unexpected TMD conditions, such as temporomandibular joint osteoarthritis, which leads to unsuccessful orthodontic treatment with reasonably unstable results.

## Temporomandibular disorders are functional disorders of the masticatory system with complex and multifactorial etiology. Different therapies are referred to in the literature for the treatment of these disorders, for instance, occlusal, pharmacological, physiotherapeutic and psychological therapies. What is your opinion about the applicability of occlusal therapy performed by means of relaxation splints in patients with temporomandibular dysfunction and in need of orthodontic treatment? (Rogério Lacerda dos Santos)

The best therapeutic strategies are chosen according to the signs and symptoms of TMD. The Japanese Society of TMD provides a treatment protocol for each type of TMD according to the following symptoms and intra-articular pathologies:

»Type I: Disorders of the masticatory muscles.
»Type II: Disorders of ligaments and soft tissues.
»Type III: Disorders of articular disc or condyle.
  - Subtype III a: Anterior disc displacement with reduction.
  - Subtype III b: Anterior disc displacement without reduction.
» Type IV: Temporomandibular joint osteoarthritis with progressive condylar resorption.

**Figure 1:**
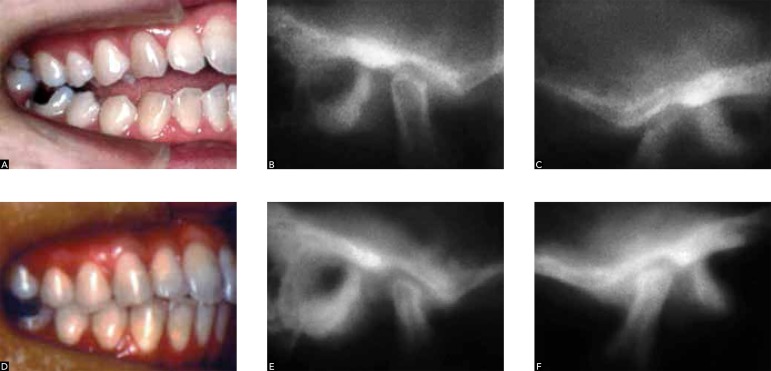
A, B, C) Pre-treatment: Abrasion of anterior teeth suggests recently developed open bite, probably originating from temporomandibular joint osteoarthritis. Special attention must be paid to the right and left condyles. D, E, F) Post-treatment: Unexpected reshaping of condyles. This phenomenon is a clinical evidence of regeneration or adaptation that can occur when stable and satisfactory occlusion is achieved by means of orthodontic/prosthetic treatment.

For types I or II, the relaxation splint is recommended to eliminate the pain in the tissues, given that it promotes decompression. Pharmacological intervention may be effective for symptomatic relief in some patients. After regression of symptoms, occlusal orthodontic treatment or occlusal treatment with prosthetic rehabilitation must be performed in cases in which the malocclusion is the cause of TMD.

As for subtype III a, the anterior repositioning splint is recommended to correct the position of the disc as well as the condyle. After repositioning the disc and the condyle, orthodontic/prosthetic treatment must be performed in order to keep the new condylar position, given that the result obtained with the therapy performed with splints is unstable and often relapses. As for types III b and IV, repositioning the disc in the condyle is considered a difficult task, therefore, it is wise not to contribute to the progression of intra-articular pathology. In this case, occlusal reconstruction may be recommended. In fact, our cases have demonstrated that progressive condylar resorption may be cured or repaired after a stable occlusion is achieved in association with normal condylar positioning.

## Based on your clinical experience, especially with regard to patients with open bite, deep bite and posterior crossbite with temporomandibular disorder, how often do relapses occur and/or temporomandibular disorders persist in these patients? Would these relapses be more related to the complex etiology of the relationship between malocclusion and TMD or to inappropriate orthodontic treatment? (Rogério Lacerda dos Santos)

This is a difficult question to answer because the relapse of malocclusion is different from the relapse of TMD. Based on my experience, the relapse of malocclusions orthodontically treated are due to deficient retention, little patient's cooperation with regard to the use of retainers, persistence of deleterious habits related to the etiology of the malocclusion (mouth breathing, tongue protrusion) and unsatisfactory treatment. Relapse of TMD occasionally occurs, however, it may be difficult to identify the reason why it occurs. It has been demonstrated that the relapse of TMD or progression of mild TMD into temporomandibular joint osteoarthritis, during and after orthodontic treatment, clearly suggests posteroinferior mandibular displacement followed by condylar resorption, in addition to Class II relationship with open bite as well as open mandibular angle, which is more severe than the original malocclusion. Based on the aforementioned considerations, it is expected that orthodontists correctly assess the patient, ensuring that the joint is healthy before orthodontic treatment onset.

## It is a great honor to have the opportunity to interview Dr. Tanne about a very interesting and relevant subject matter: teeth bank. Researches on tooth cryopreservation and reimplantation have been brilliantly conducted at the University of Hiroshima and will certainly contribute not only to dental rehabilitation, but also to improve patients' quality of life. Professor Tanne, could you briefly explain how it all started and describe the current status of the University of Hiroshima teeth bank? (Matheus Melo Pithon)

I greatly appreciate your interest in tooth cryopreservation. Tooth transplantation is covered by health insurance in Japan and it is considered a conventional treatment method employed to replace lost teeth. However, tooth transplantation must be carried out immediately after tooth extraction. Tooth extraction is well accepted and is part of many orthodontic treatment plans, for instance, in cases of great tooth/bone discrepancy. Transplantation is not necessary in the majority of these cases, yet the extracted teeth are discarded as hospital waste. In this context, we have developed an advanced system of cryopreservation under magnetic field called CAS (Cells Alive System). To validate the CAS system, we initially conducted an *in vitro *study with a culture of fibroblasts originating from the periodontal ligament in order to exam the survival and proliferation rates of the cryopreserved cells after thawing. The results demonstrated survival and proliferation rates that were satisfactory for transplantation. Afterwards, an *in vitro *study was conducted to assess the feasibility of transplanting cryopreserved teeth in rats. Upper central incisors that had been immediately reimplanted served as control. The results did not reveal the development of ankylosis and the root resorption rate did not present any difference among the teeth that served as control. Subsequently, with permission of the Japanese Ministry of Health, we transplanted a cryopreserved tooth in a 28-year-old female patient, the first experiment of this nature in the world. The results were excellent, with significant regeneration of periodontal tissues. So far, there are more than 1,800 cryopreserved teeth in our teeth bank. Among these, approximately 130 teeth have returned to their original patients with a success rate of 95%. Therefore, it is reasonable to conclude that our teeth storage system is extremely useful to preserve teeth that have been extracted due to orthodontic indication. Additionally, the application of this technology in tissue engineering, particularly with regard to the preservation of other types of tissues and cells, such as stem cells, seems to be highly promising. In fact, we have demonstrated that cryopreserved mesenchymal stem cells induce bone repair in bone defects created in growing rats.

**Figure:**
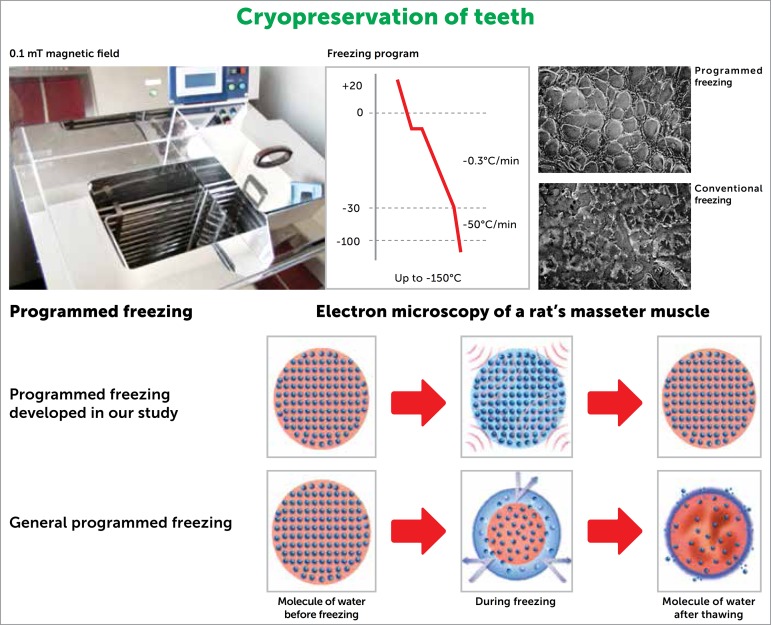


## Professor, could you briefly explain the cellular mechanism involved in the preservation of a tooth? Are there any studies regarding orthodontic movement in sites that have received cryopreserved cells? (Matheus Melo Pithon)

In the magnetic field obtained with the CAS freezer, the molecules of water inside the cells vibrate and are uniformly aligned. Therefore, at thawing, they do not destroy the cell wall, whereas in the conventional freezer the molecules of water freeze, forming clusters that, when undone at thawing, destroy the cell wall. This fact results in low survival rate and low cell proliferation, which leads to unsuccessful transplantation due to poor tissue regeneration.

To assess the metabolic activity of mesenchymal stem cell regenerated bones of Beagle dogs, we conducted a study in which teeth were subjected to movement within the new regenerated bone tissue. Tooth movement rate was similar to the control groups, thus suggesting an equivalent metabolic function.

**Figure:**
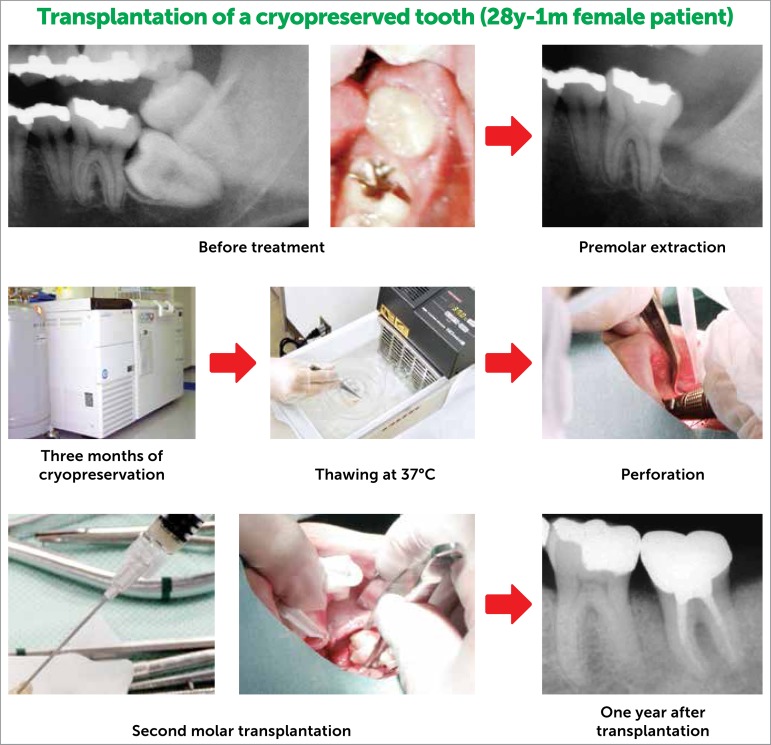


## Do you believe that, in the future, human teeth will become organs eligible for donation, especially in countries that do not have access to modern technologies? Do you believe that it will be possible to "recreate teeth"? Is it possible to implement the technology of teeth bank in Brazil? (Matheus Melo Pithon)

Our storage system is strictly maintained to preserve and reimplant the teeth from and to the same patient. Several infectious diseases can exist in the oral cavity where they can be passed on in case transplantation is carried out in a distinct receptor. This is a basic principle behind our teeth bank. With regard to your second question, we have already implemented our system of teeth bank at the Medical School of Taipei University, in Taiwan. For this reason, it is perfectly possible to implement the same system in Brazil, provided that some basic principles are met. I would like to request that everybody seek new innovations in favor of Dentistry and Orthodontics. Finally, I would like to express my sincere thanks to Dental Press Journal of Orthodontics, all the interviewers as well as readers.

